# Trajectories of 12-Month Usage Patterns for Two Smoking Cessation Websites: Exploring How Users Engage Over Time

**DOI:** 10.2196/10143

**Published:** 2018-04-20

**Authors:** Jonathan B Bricker, Vasundhara Sridharan, Yifan Zhu, Kristin E Mull, Jaimee L Heffner, Noreen L Watson, Jennifer B McClure, Chongzhi Di

**Affiliations:** ^1^ Fred Hutchinson Cancer Research Center Seattle, WA United States; ^2^ Department of Psychology University of Washington Seattle, WA United States; ^3^ Kaiser Permanente Washington Health Research Institute Seattle, WA United States

**Keywords:** engagement, trajectories, eHealth, websites, tobacco, smoking, acceptance and commitment therapy, smokefree.gov, patient participation, telemedicine, tobacco use cessation, smoking cessation

## Abstract

**Background:**

Little is known about how individuals engage with electronic health (eHealth) interventions over time and whether this engagement predicts health outcomes.

**Objective:**

The objectives of this study, by using the example of a specific type of eHealth intervention (ie, websites for smoking cessation), were to determine (1) distinct groups of log-in trajectories over a 12-month period, (2) their association with smoking cessation, and (3) baseline user characteristics that predict trajectory group membership.

**Methods:**

We conducted a functional clustering analysis of 365 consecutive days of log-in data from both arms of a large (N=2637) randomized trial of 2 website interventions for smoking cessation (WebQuit and Smokefree), with a primary outcome of 30-day point prevalence smoking abstinence at 12 months. We conducted analyses for each website separately.

**Results:**

A total of 3 distinct trajectory groups emerged for each website. For WebQuit, participants were clustered into 3 groups: 1-week users (682/1240, 55.00% of the sample), 5-week users (399/1240, 32.18%), and 52-week users (159/1240, 12.82%). Compared with the 1-week users, the 5- and 52-week users had 57% higher odds (odds ratio [OR] 1.57, 95% CI 1.13-2.17; *P*=.007) and 124% higher odds (OR 2.24, 95% CI 1.45-3.43; *P*<.001), respectively, of being abstinent at 12 months. Smokefree users were clustered into 3 groups: 1-week users (645/1309, 49.27% of the sample), 4-week users (395/1309, 30.18%), and 5-week users (269/1309, 20.55%). Compared with the 1-week users, 5-week users (but not 4-week users; *P*=.99) had 48% higher odds (OR 1.48, 95% CI 1.05-2.07; *P*=.02) of being abstinent at 12 months. In general, the WebQuit intervention had a greater number of weekly log-ins within each of the 3 trajectory groups as compared with those of the Smokefree intervention. Baseline characteristics associated with trajectory group membership varied between websites.

**Conclusions:**

Patterns of 1-, 4-, and 5-week usage of websites may be common for how people engage in eHealth interventions. The 5-week usage of either website, and 52-week usage only of WebQuit, predicted a higher odds of quitting smoking. Strategies to increase eHealth intervention engagement for 4 more weeks (ie, from 1 week to 5 weeks) could be highly cost effective.

**Trial Registration:**

ClinicalTrials.gov NCT01812278; https://www.clinicaltrials.gov/ct2/show/NCT01812278 (Archived by WebCite at http://www.webcitation.org/6yPO2OIKR)

## Introduction

Electronically delivered health interventions (or eHealth interventions), such as websites and mobile apps, have been successful methods of health behavior change [1-4]. In this body of research, people who engage more with eHealth interventions tend to have better treatment outcomes [5]. However, while eHealth intervention engagement is usually measured with simple counts of the number of log-ins and modules completed [5], little is known about how users engage with eHealth interventions *over time* and whether those temporal patterns predict better treatment outcomes. In the educational literature, a well-documented finding is that learning new material becomes more effective when it occurs over a longer period of time as opposed to over a short period of time [6]. This process, called spaced practice, works by way of increasing variability in learning and remembering new information [7].

Websites and mobile apps for health behavior change are usually available for participants to use at will, which results in high variations of individual usage patterns, or usage trajectories, over time. For example, some users may follow a trajectory of logging in several times within the first few days of starting an intervention and then never return. Others may follow a trajectory where they log in consistently and then gradually taper off. And other users may follow a trajectory where they consistently log in over the course of many months. It is possible that some groups of individuals follow unique usage trajectories over time that are associated with differential health outcomes. For example, people who log in consistently over the course of many months might have positive health outcomes because they have consistently benefited from the information and skills presented in the intervention. Alternatively, consistent log-ins may be a marker of ongoing challenges and struggles to change a health behavior, and thus may indicate poorer treatment outcomes. Since we do not know which trajectories of use predict successful behavior change, studying distinct groups of usage trajectories that people follow can help us identify which usage patterns are beneficial and make recommendations for future program use. This will help inform the design of eHealth interventions to improve successful behavior change.

Within the social and behavioral sciences, identifying usage trajectories has been applied for several decades to understanding behavior patterns over time [8-11]. More recently, a few studies have analyzed usage trajectories for eHealth interventions. One study examined 8-week usage trajectories of a diabetes management mobile app. The study found 3 distinct trajectories of usage and described the clusters of people following these trajectories as minimal users, intermittent waning users, and consistent users [12]. However, the study was limited by a small sample size (N=84), as well as short duration (8 weeks), and whether the trajectories predicted health outcomes was not reported. Other research identified 5 distinct usage trajectories of a short message service (SMS) text-messaging–based smoking cessation program over 5 weeks, namely high engagement, increasing engagement, rapid decrease, delayed decrease, and low engagement [13]. The study found that the high engagement and increasing engagement groups were more likely than the other groups to be abstinent over the course of 5 weeks.

If eHealth intervention usage trajectories that predict health outcomes can be identified, understanding the groups of individuals who tend to follow more or less successful trajectories is an important next step. This would reveal the qualities of individuals who are likely to have engagement patterns that are related to successful and unsuccessful outcomes. Knowing these baseline characteristics might allow researchers and intervention designers to tailor eHealth interventions to users’ unique challenges, needs, and limitations. While studies have found that being a woman, being older, and having a higher education are generally consistent predictors of greater eHealth intervention usage [14-17], very little is known about the user characteristics that are associated with different patterns of use over time. To our knowledge, only 1 study has examined this question [12] and found that being female and having higher baseline motivation were associated with more consistent log-in trajectories.

Using the example of smoking cessation websites, in this study we aimed to determine (1) distinct groups of log-in trajectories, (2) their prediction of the smoking cessation outcome, and (3) baseline user characteristics that are associated with different usage trajectory groups. The overall goal was to advance the study of analytic methods of user engagement and, ultimately, the design of more effective interventions that are tailored to users and their longitudinal patterns of engagement. To accomplish these aims, in this study we analyzed 365 consecutive days of log-in data from both arms of a large (N=2637), 2-arm randomized trial of website interventions for smoking cessation (NCT01812278).

## Methods

### Participants

As described in the main outcome article for the trial [18], we recruited participants (N=2637) from across the United States to participate in a study comparing 2 Web-delivered smoking cessation programs. Participants were recruited between March 24, 2014 and August 11, 2015. To be eligible for the study, participants had to be adult smokers in the United States (≥18 years of age), smoking at least 5 cigarettes daily, motivated to quit in the next 30 days, and have internet access. The 2637 participants were assigned to 1 of 2 Web-based smoking cessation interventions using stratified black randomization (on smoking frequency, education, and sex): WebQuit (n=1319; experimental arm) [18] or Smokefree (n=1318; control arm) [19].

### Smoking Cessation Interventions

Participants accessed their assigned website with a unique username and password. For the first 4 weeks, all participants in both programs could opt to receive up to 4 short daily tips via SMS text messaging or email, which were designed to increase engagement. Participants were free to use their assigned program as they wished for 1 year from the date of enrollment.

The WebQuit program was based on acceptance and commitment therapy (ACT) [20], an approach that teaches skills to smokers to let their urges pass without smoking. The program had 4 parts. Step 1, Make a Plan, enabled users to develop a personalized quit plan, identify smoking triggers, learn about US Food and Drug Administration (FDA)-approved cessation medications, and upload a photo of their inspiration to quit (ACT processes: Values and Committed Action). Step 2, Be Aware, contained 3 exercises to illustrate the problems with trying to control thoughts, feelings, and physical sensations rather than allowing them to come and go (ACT process: Creative Hopelessness). Step 3, Be Willing, contained 8 exercises to help users practice allowing thoughts, feelings, and physical sensations that trigger smoking (ACT processes: Willingness, Being Present, and Cognitive Defusion). Step 4, Be Inspired, contained 15 exercises to help participants identify deeply held values inspiring them to quit smoking and to exercise self-compassion in response to smoking lapses (ACT processes: Values and Self-as-Context). The program also prompted users to track smoking, cessation medications, and practice of ACT skills. Tracking results were displayed graphically along with the user’s inspiration for quitting and badges earned for program use. Participants could log in and use the program as much as they liked.

For the control arm, we hosted a secured private version of the US National Cancer Institute’s Smokefree.gov site. This intervention was also named WebQuit so that participants would be blinded to group assignment. Smokefree follows the US clinical practice guidelines [21] and provides standard treatment that teaches skills to smokers to avoid urges. Users were able to navigate through all pages of the website at any time, and there were no restrictions on the order in which they could view the content. Smokefree had 3 main sections: Quit Today, Preparing to Quit, and Smoking Issues. The Quit Today section had 7 pages of content that provided tips for the quit day, staying smoke-free, and dealing with cravings. The section also provided information on withdrawal, benefits of quitting, and FDA-approved cessation medications. The Prepare to Quit section had 7 content pages providing information on various reasons to quit, what makes quitting difficult, how to make a quit plan, and using social support during a quit attempt. The Smoking Issues section provided 5 pages on health effects of smoking and quitting, depression, stress, secondhand smoke, and coping with the challenges of quitting smoking for the lesbian, gay, bisexual, and transgender community. The section also contained 5 quizzes that provided feedback about level of depression, stress, nicotine dependence, nicotine withdrawal, and secondhand smoke, as well as tips for coping with them.

### Measures

#### Baseline Characteristics

At baseline, participants reported on demographics, alcohol use, smoking history, and whether they had a partner and friends who smoked. We measured nicotine dependence with all 6 items of the Fagerström Test for Nicotine Dependence (FTND) [22]. Participants also filled out the Commitment to Quitting Scale [23], which has 8 items measuring participants’ motivation to stay abstinent (example item, “I’m willing to put up with whatever discomfort I have to in order to quit smoking.”). The scale, which has been used in multiple smoking cessation trials [18,24], has been shown to have good reliability and validity [23]. We screened participants for mental health conditions including depression (Center for Epidemiologic Studies Depression scale) [25], generalized anxiety (Generalized Anxiety Disorder 7-item scale) [26], panic disorder (Autonomic Nervous System Questionnaire) [27], posttraumatic stress disorder (PTSD; PTSD Checklist) [28], and social anxiety (mini-Social Phobia Inventory) [29]. We included the results as covariates and predictors, since prior research has shown that mental health symptoms are a predictor of engagement in eHealth interventions [30,31].

#### Engagement

For each participant, we recorded time- and date-stamped log file records of each page opening. For this analysis, we used a binary measure indicating whether each participant logged in at least once each day (ie, had at least one page opening recorded in the log file data). Using this method, we obtained for each participant a 0/1 code for each day for 365 days from the date of randomization.

#### Cessation Outcome

The primary outcome of the study was self-reported 30-day point prevalence abstinence (ie, no smoking at all in the past 30 days) at 12-month follow-up. Self-reported smoking or abstinence is a standard method for assessing the efficacy of Web-delivered interventions [32]. The Society for Research on Nicotine and Tobacco Subcommittee on Biochemical Verification has suggested that biochemical confirmation is not necessary in population-based studies with no face-to-face contact and in studies where data are collected through the Web, telephone, or mail because of low demand characteristics of these studies [33,34].

### Statistical Analyses

To determine distinct groups of log-in trajectories for each website, we used a functional clustering approach consisting of 3 steps: (1) presmoothing the binary daily engagement time series; (2) conducting functional principal component analysis [35], a dimension reduction procedure to summarize each participant’s log-in trajectory by low-dimensional functional principal component scores; and (3) applying the clustering large applications algorithm [36] to the derived functional principal component scores. This procedure does not rely on any assumptions on the shapes of trajectories and is capable of handling large datasets and complex missing data patterns. We determined the total number of trajectories for each website using predictive strength [37], which is a statistical criterion to assess how many groups can be predicted from the data and how well. We obtained each study participant’s log-in trajectory by transforming longitudinal sequences of log-in time stamps into a binary time series indicating log-in occurrence each day. Note that we chose not to use latent class growth curve approaches that have been used in other eHealth intervention engagement studies [12,13] because these methods do not handle very densely recorded longitudinal data without substantial data reduction (eg, reducing data into weekly or monthly log-in counts per participant) and often rely on restrictive assumptions on the shapes of trajectories.

After determining distinct trajectory clusters, we applied logistic regression models to investigate the associations between the trajectory clusters and the smoking cessation outcome. Both unadjusted and covariate-adjusted regression models were fitted. For covariate-adjusted models, we selected variables by stepwise Akaike information criterion (AIC) in both backward and forward directions. Covariates considered for adjustment were the baseline characteristics described above in the Measures subsection, including commitment to quit smoking, to control for participant characteristics that may confound any association with cessation outcomes. Finally, to identify baseline user characteristics associated with trajectory membership, we applied multinomial logistic regression models with baseline covariates as predictors and the log-in trajectory clusters as outcome. We selected variables in the final multivariate model via a stepwise AIC procedure from a pool of candidate baseline covariates that had a univariate association with log-in trajectory clusters.

## Results

### Description of Sample

Table 1 shows the baseline demographics and participant characteristics in both the WebQuit and Smokefree arms. Overall, participants were on average 46 years old, about 80% were female, about 80% were white, about 52% were employed, and about 72% had greater than high school education.

**Table 1:** Summary of baseline characteristics of participants from both WebQuit and Smokefree arms, by log-in trajectories. LGBT: lesbian, gay, bisexual, and transgender; FTND: Fagerström Test for Nicotine Dependence; PTSD: posttraumatic stress disorder.

Participant characteristics		WebQuit (n=1240)				Smokefree (n=1309)			
		1-week users (n=682)	5-week users (n=399)	52-week users (n=159)	Overall	1-week users (n=645)	4-week users (n=395)	5-week users (n=269)	Overall
Age (years), mean (SD)		44.6 (13.6)	47.4 (12.5)	51.4 (12.6)	46.4 (13.3)	45.4 (13.2)	46.0 (13.8)	48.1 (12.8)	46.2 (13.3)
Male, n (%)		149 (21.8)	67 (16.8)	32 (20.1)	248 (20.0)	133 (20.6)	90 (22.8)	49 (18.2)	272 (20.8)
Married, n (%)		263 (38.6)	174 (43.6)	56 (35.2)	493 (39.8)	234 (36.3)	140 (35.4)	89 (33.1)	463 (35.4)
Working, n (%)		354 (51.9)	212 (53.1)	83 (52.2)	649 (52.3)	362 (56.2)	196 (49.6)	118 (43.9)	676 (51.6)
High school or less, n (%)		204 (29.9)	98 (24.6)	42 (26.4)	344 (27.7)	185 (28.7)	107 (27.1)	70 (26.0)	362 (27.7)
LGBT, n (%)		63 (9.2)	32 (8.0)	14 (8.8)	109 (8.8)	63 (9.8)	42 (10.6)	30 (11.2)	135 (10.3)
White, n (%)		558 (81.2)	323 (81.0)	123 (77.4)	1004 (81.0)	530 (82.2)	321 (81.3)	218 (81.0)	1069 (81.7)
Hispanic, n (%)		52 (7.6)	34 (8.5)	6 (3.8)	92 (7.42)	52 (8.1)	42 (10.6)	27 (10.0)	121 (9.2)
Any quit attempt in last 12 months, n (%)		269 (42.0)	165 (43.9)	70 (46.1)	504 (43.1)	285 (45.7)	169 (44.5)	121 (46.9)	575 (45.6)
FTND score, mean (SD)		5.68 (2.19)	5.54 (2.18)	5.58 (2.21)	5.62 (2.19)	5.70 (2.10)	5.71 (2.17)	5.33 (2.32)	5.63 (2.17)
**Smoking characteristics**									
	Half a pack or more, n (%)	539 (79.0)	313 (78.4)	128 (80.5)	980 (79.0)	523 (81.1)	313 (79.2)	195 (72.5)	1031 (78.8)
	>10 years, n (%)	530 (77.7)	327 (82.0)	139 (87.4)	996 (80.3)	509 (78.9)	310 (78.5)	223 (82.9)	1042 (79.6)
	Partner smokes, n (%)	459 (67.3)	279 (69.9)	115 (72.3)	853 (68.8)	454 (70.4)	270 (68.4)	200 (74.3)	924 (70.6)
	No. of friends who smoke, mean (SD)	2.2 (1.6)	2.1 (1.6)	2.1 (1.7)	2.2 (1.6)	2.3 (1.6)	2.1 (1.6)	2.3 (1.7)	2.2 (1.6)
Commitment score, mean (SD)		4.01 (0.74)	3.97 (0.75)	3.94 (0.77)	3.99 (0.75)	4.01 (0.77)	3.96 (0.73)	4.04 (0.82)	4.00 (0.77)
**Mental health measures, n (%)**									
	Depression	400 (59.0)	208 (52.3)	80 (50.3)	688 (55.7)	374 (58.4)	208 (52.9)	149 (55.8)	731 (56.2)
	Anxiety	246 (36.4)	123 (31.1)	41 (25.8)	410 (33.3)	238 (37.0)	130 (33.0)	93 (34.6)	461 (35.3)
	Social anxiety	197 (29.0)	123 (30.8)	37 (23.4)	357 (28.9)	191 (29.7)	125 (31.7)	88 (32.8)	404 (30.9)
	Panic	304 (49.0)	167 (47.6)	58 (42.3)	529 (47.7)	292 (49.7)	173 (49.3)	117 (48.3)	582 (49.3)
	PTSD	368 (54.3)	207 (52.1)	72 (45.3)	647 (52.4)	357 (55.4)	187 (47.5)	143 (53.2)	687 (52.6)
Hazardous alcohol use, n (%)		83 (12.4)	38 (9.7)	13 (8.6)	134 (11.0)	82 (13.0)	36 (9.5)	23 (8.7)	141 (11.1)
Alcohol or drug abuse, n (%)		36 (5.3)	20 (5.0)	10 (6.3)	66 (5.32)	37 (5.7)	31 (7.8)	14 (5.2)	82 (6.3)

There were no baseline differences between treatment arms on how often participants had used the internet in the last 30 days (χ^2^_2, n=__2495_=2.3, *P*=.32). Fewer than half (about 42%) of the participants had made a quit attempt in the last year, and about 80% of the sample had been smoking for more than 10 years, with an average FTND score of 5.6 (moderate nicotine dependence). The data retention rate was 87.56% (2309/2637) and did not differ between arms.

### Description of Distinct Groups of Trajectories

**Figure 1:**
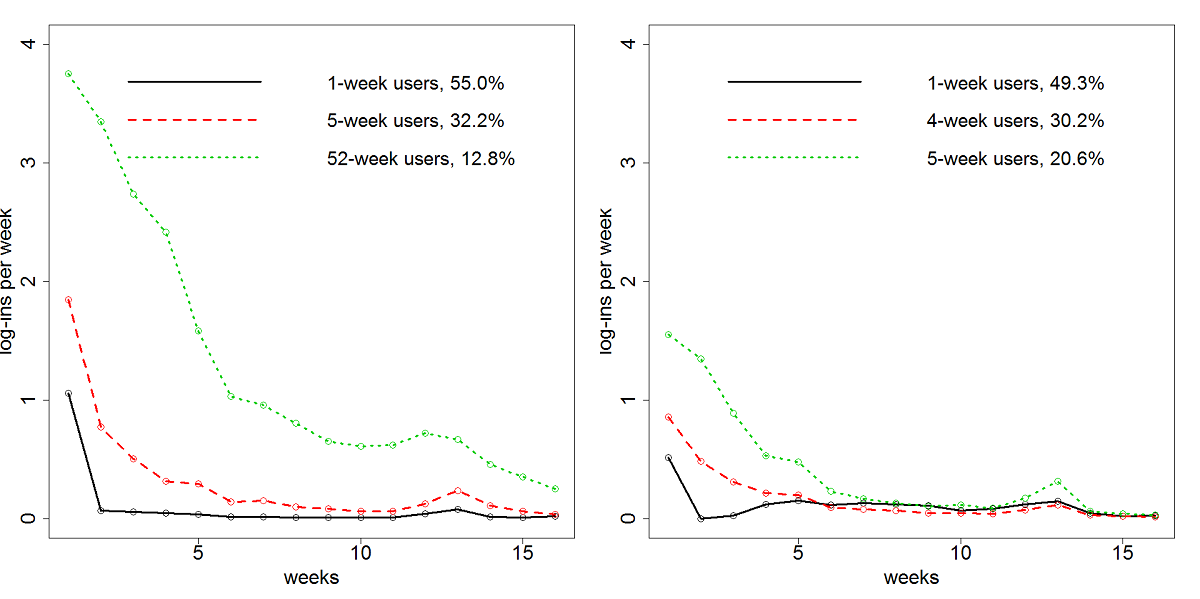
Average weekly log-in trajectory for each cluster from the (left) WebQuit (n=1240) arm and (right) Smokefree (n=1309) arm for first 16 weeks of use.

The functional clustering analysis of 52 weeks of log-ins revealed 3 distinct groups of trajectories for each of the intervention websites. Figure 1 shows log-in patterns for the first 16 weeks for WebQuit (left) and for Smokefree (right). The trajectories were easiest to visualize for the first 16 weeks of use. However, Multimedia Appendix 1 shows the full 52 weeks for reference. For the WebQuit website (Figure 1, left), the first trajectory group (682/1240, 55.00% of sample) logged at least one day in the first week and then had almost no log-ins after that. They were termed 1-week users. The second trajectory group (399/1240, 32.18% of sample) logged in an average of 1.8 days in the first week, 0.8 days in the second week, once every 3 weeks until week 5, and had very sporadic log-ins in week 6 and beyond. They were termed 5-week users. The third trajectory group (159/1240, 12.82% of sample) logged in an average of 3.7 days in the first week, 3.3 days in the second week, 2.7 days in the third week, 2.4 days in the fourth week, 1.6 days in week 5, once in week 6, and then on average once every month starting in week 7 and continuing in this pattern until week 52. They were termed 52-week users.

For the Smokefree website (Figure 1, right), the first trajectory group (645/1309, 49.27% of sample) logged in less than once on average in the first week and then had almost no log-ins after that. As with WebQuit, they were termed 1-week users. The second trajectory group (395/1309, 30.18% of sample) logged in once in week 1, every other week until week 4, and then had almost no log-ins after that. They were termed 4-week users. The third trajectory group (269/1309, 20.55% of sample) logged in an average of 1.5 days in weeks 1 and 2, once in week 3, every other week over the period of weeks 4 to 5, and then had almost no log-ins after that. They were termed 5-week users. Note also that in both intervention arms, there was a pattern of a spike in log-ins at week 12, corresponding to the invitation to complete the 12-week outcome survey that, while completely independent of the interventions, likely triggered some users to engage with their assigned intervention website.

### Trajectory Membership Prediction of Smoking Cessation Outcome

Table 2 shows each intervention arm’s trajectory group membership as a predictor of 30-day point prevalence abstinence at the 12-month follow-up. For WebQuit, abstinence rates for these 3 trajectory groups were 116/562 (20.6%) for 1-week users, 100/370 (27.0%) for 5-week users, and 51/149 (34.2%) for 52-week users. Compared with 1-week users, 5-week users had 57% higher odds (OR 1.57, 95% CI 1.13-2.17; *P*=.007) of being abstinent at 12 months, and 52-week users had 124% higher odds (OR 2.24, 95% CI 1.45-3.43; *P*<.001) of being abstinent at 12 months. These models adjusted for the baseline covariates selected as outlined in the Methods section and included smoking half a pack or more, the commitment to quitting score, and screening positive for panic disorder. Descriptively, for Smokefree, abstinence rates for the 3 trajectory groups were 139/562 (24.7%) for 1-week users, 85/349 (24.4%) for 4-week users, and 81/252 (32.1%) for 5-week users.

**Table 2:** Logistic regression models predicting 12-month smoking cessation outcome by groups of engagement trajectories and other covariates^a^.

Arm and covariate		Odds ratio	95% CI	*P* value
**WebQuit**				
	5-week users	1.57	1.13-2.17	.007
	52-week users	2.24	1.45-3.43	<.001
	Half a pack or more	0.58	0.41-0.82	.002
	Commitment	1.69	1.37-2.10	<.001
	Panic^b^	0.75	0.55-1.01	.06
**Smokefree**				
	4-week users	1.00	0.73-1.37	.99
	5-week users	1.48	1.05-2.07	.02
	High school or less	0.69	0.50-0.94	.02
	Smoking >10 years	0.76	0.55-1.06	.10
	Smokes within 5 minutes of waking	0.75	0.57-0.99	.05
	Commitment	1.71	1.41-2.07	<.001
	Partner smokes	0.62	0.47-0.84	.001

^a^Reference group: 1-week users. Only significant predictors have been included in this table for ease of reading.

^b^Refers to whether participants screened positive for panic disorder.

**Table 3:** Multinomial logistic regression results predicting log-in trajectory cluster membership from baseline characteristics^a^.

Arm, cluster, and characteristic			Odds ratio	95% CI
**WebQuit**				
	**5-week users**			
		Smoking >10 years	1.27	0.93-1.75
		Not anxious^b^	1.25	0.96-1.64
	**52-week users**			
		Smoking >10 years	1.90	1.14-3.14
		Not anxious	1.56	1.06-2.33
**Smokefree**				
	**4-week users**			
		Less than half a pack	1.16	0.85-1.61
		Unemployed	1.33	1.03-1.72
		No posttraumatic stress disorder^c^	1.43	1.11-1.85
	**5-week users**			
		Less than half a pack	1.72	1.23-2.44
		Unemployed	1.79	1.33-2.38
		No posttraumatic stress disorder	1.16	0.88-1.56

^a^Reference group: 1-week users. Only significant predictors have been included in this table for ease of reading.

^b^Refers to screening negative for generalized anxiety disorder.

^c^Refers to screening negative for posttraumatic stress disorder.

Compared with 1-week users, 4-week users were not more likely to be abstinent at 12 months (OR 1.00, 95% CI 0.73-1.37; *P*=.99), but 5-week users had 48% higher odds of being abstinent (OR 1.48, 95% CI 1.05-2.07; *P*=.02). This analysis adjusted for selected baseline covariates of education, smoking more than 10 years, smoking within 5 minutes of waking, commitment to quitting, and whether one has a partner who smokes.

### Baseline Characteristics Predicting Trajectory Membership

Since the groups of trajectories were different across the 2 arms, we explored the baseline characteristics predicting membership in the groups for the 2 arms separately. For WebQuit, baseline characteristics associated with trajectory membership were age, smoking for at least the past 10 years, screening positive for depression, and screening positive for anxiety (all *P*<.05; results not shown). Controlling for the impact of related covariates, the adjusted multivariate regression model selected by stepwise AIC procedure showed that smoking for at least the past 10 years and screening negative for anxiety each, respectively, predicted a 90% higher odds (OR 1.90, 95% CI 1.14-3.14) and a 56% higher odds (OR 1.56, 95% CI 1.06-2.33) of being a 52-week user (compared with being a 1-week user) (Table 3). Since smoking history is partly a reflection of one’s age, and the variables age, smoking history, and anxiety were correlated with each other, when we calculated a model containing age (categorized by decade), only age emerged as a significant predictor (see Multimedia Appendix 2).

For Smokefree, the baseline characteristics associated with trajectory membership in univariate analysis were being unemployed, smoking less than half a pack per day, and screening as not having PTSD (all *P*<.05; results not shown). Controlling for the impact of related covariates, the multivariate regression model showed that smoking less than half a pack per day predicted a 72% higher odds (OR 1.72, 95% CI 1.23-2.44) of being a member of the 5-week group, compared with the 1-week user group (Table 3). Being unemployed predicted a 79% higher odds (OR 1.79, 95% CI 1.33-2.38) of being a member of the 5-week user group relative to the 1-week group. Screening negative for PTSD predicted 43% higher odds (OR 1.43, 95% CI 1.11-1.85) of being a member of the 4-week user group relative to the 1-week user group. There was no evidence in either sample that sex predicted trajectory membership (all *P*>.05).

## Discussion

### Principal Findings

To our knowledge, this was one of few studies to analyze usage trajectories of eHealth interventions and examine the association between trajectory group membership and health outcomes [12,13]. The study found (1) 3 distinct groups of log-in trajectories for 2 Web-delivered interventions for smoking cessation, (2) that these trajectory groups differentially predicted smoking outcomes at 12 months, and (3) that certain user characteristics are associated with membership in certain trajectory groups. A 5-week usage of either website, and 52-week usage only of WebQuit, predicted a higher odds of quitting smoking. In general, the WebQuit intervention had a greater number of weekly log-ins within each of the 3 trajectory groups as compared with those of the Smokefree intervention. These major results are synthesized and interpreted in greater detail in this discussion.

### Usage Trajectories and Health Outcomes

Regarding the first trajectory group, half the participants in both arms were 1-week users, which is a significant concern because they were the least likely to abstain from smoking at 12 months. Thus, it is imperative to learn why a participant would have almost no log-ins after a single week of use. User-centered design research, including laboratory observations and diary studies, could help elucidate the qualities of the intervention that cause an individual to discontinue use of the website. These individuals might benefit from a more intensive intervention, an eHealth intervention that uses a different treatment model, or one that is *not* eHealth (eg, individual telephone coaching). Regarding the second trajectory group, 5-week users were more likely to quit smoking in the WebQuit intervention (as well as for Smokefree, which had 5-week users as its third trajectory group). These results suggest that strategies to increase eHealth intervention engagement for 4 more weeks (ie, from 1 week to 5 weeks) could be highly cost effective. Example strategies worth testing include (1) proactive check-ins (via text message or phone calls) from staff about progress with the website, (2) daily automated text messages notifying the user of new content now available on the website, (3) rewards for each day’s use of the website with badges or redeemable prizes, and (4) a 5-week challenge that shows other users’ daily log-in progress toward the goal of 5 weeks of usage.

Regarding the third trajectory group, each intervention website had distinct log-in patterns that are likely explained by differing website structures. For Smokefree, this group was the 5-week users. The fact that they had almost no log-ins at 5 weeks and beyond is likely a reflection of Smokefree’s structure—an informational resource for users, functioning like reference material. Thus, 5 weeks may be sufficient time for a user to glean all needed information from Smokefree and apply it appropriately to quitting smoking, as they had 48% higher odds of quitting smoking (compared with 1-week users). For WebQuit, this group was the 52-week users, who had 124% higher odds of quitting smoking (compared with 1-week users). Their much longer-term engagement is likely a reflection of WebQuit’s structure—a step-by-step skills-based program that includes tracking progress with urges and smoke-free days. This program structure may have encouraged long-term, spaced skills practice [6], which may have contributed to the 34% 12-month quit rates observed in WebQuit’s third trajectory group. In general, the findings for both websites’ third trajectory group suggest that consistent use of each program over time is prognostic of a better health outcome, which is contrary to the notion that consistent log-ins may be a marker of ongoing challenges and struggles to change a health behavior. E-intervention design should thus focus on methods to encourage engagement over time, which may include strategies similar to those suggested above.

### Personal Characteristics and Usage Trajectories

The impact of personal characteristics on usage trajectories appeared to vary by intervention. Specifically, WebQuit users who had smoked for at least 10 years were more likely to be 5-week users and nearly twice as likely to be 52-week users than 1-week users. However, smoking history differences may be a reflection of age: users aged 50 years and over were over 8 times more likely to be 52-week users. This finding is consistent with past research showing that being older is a predictor of higher eHealth use [14-17], even though it was found only for WebQuit, not Smokefree, in this analysis. On the other hand, participants who screened positive for a mental health condition in either website (PTSD in Smokefree, and anxiety or depression in WebQuit) were more likely to be 1-week users, which suggests the need develop strategies to promote longer-term engagement for people with mental health disorders. There was no evidence in this study that sex predicted trajectory membership. Nonetheless, we recommend that future research examine many subgroup differences (eg, sex, race, age) in eHealth intervention trajectories as research on this model methodology expands to a wide variety of populations. Overall, these analyses suggest a need for further research on what baseline factors might predict different usage trajectories, and therefore inform the development of tailored interventions that facilitate long-term, consistent engagement, based on an individual’s specific baseline characteristics.

### Limitations and Future Directions

The study had several key limitations. First, we tested only 2 websites, and both were focused on smoking cessation; thus, future research should examine the extent to which results generalize to other behaviors and to other types of eHealth interventions. Second, cessation outcome data were self-reported for reasons stated in the Methods. Remote biochemical validation of smoking cessation would have introduced biases, including low response rates, prohibitive cost, challenges with confirming the identity of the person providing the sample, and inability to confirm abstinence beyond 24 hours [33,34].

### Conclusions

In general, the WebQuit intervention had a greater number of weekly log-ins within each of the 3 trajectory groups as compared with those of the Smokefree intervention. The 1-, 4-, and 5-week usage of websites may be common patterns of how people engage in eHealth interventions over time. The 5-week usage of either website, and 52-week usage only of WebQuit, predicted a higher odds of quitting smoking. Strategies to increase eHealth intervention engagement for 4 more weeks (ie, from 1 week to 5 weeks) could be highly cost effective.

## Appendix

Multimedia Appendix 1 Average weekly log-in trajectory each for cluster from the WebQuit (n=1240) arm and Smokefree (n=1309) arm for 52 weeks of use.

Multimedia Appendix 2 Age as dichotomous variable (reference: <30, age not selected in SmokeFree arm), odds ratios reported with 1-week users as comparison group.

## Abbreviations

ACT: acceptance and commitment therapy
AIC: Akaike information criterion
eHealth: electronic health
FDA: Food and Drug Administration
FTND: Fagerström Test for Nicotine Dependence
PTSD: posttraumatic stress disorder
SMS: short message service
