# Supplementary figures and images for: Trajectories of 12-Month Usage Patterns for Two Smoking Cessation Websites: Exploring How Users Engage Over Time
Source: J Med Internet Res. 2018 Apr 20;20(4):e10143. doi: 10.2196/10143 (PMC5935807; doi:10.2196/10143)

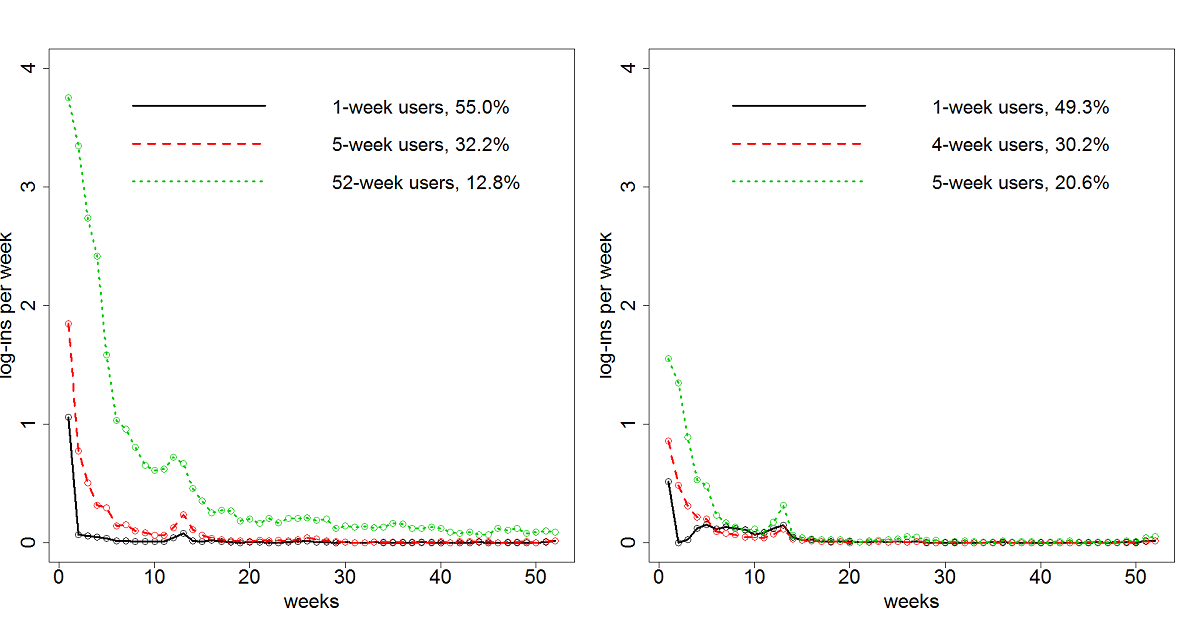

Supplement: Multimedia Appendix 1 [file jmir_v20i4e10143_app1.png]
