# Supplementary material for: Trajectories of 12-Month Usage Patterns for Two Smoking Cessation Websites: Exploring How Users Engage Over Time
Source: J Med Internet Res. 2018 Apr 20;20(4):e10143. doi: 10.2196/10143 (PMC5935807; doi:10.2196/10143)
Supplement: Multimedia Appendix 2 [file jmir_v20i4e10143_app2.pdf]

Supplementary Table: Age as dichotomous variable (reference: <30, age not selected in SmokeFree arm), OR reported with one-week users as comparison group.

	WebQuit				SmokeFree		
		OR	95% CI			OR)	95% CI
Five week users				Four week users			
	Age 30-39	1.48	(0.94,2.35)		Less than half pack	1.16	(0.85,1.61)
	Age 40-49	1.86	(1.19,2.90)		Unemployed	1.33	(1.03,1.72)
	Age 50+	2.05	(1.37,3.07)		No PTSD[a]	1.43	(1.11,1.85)
Fifty-two week users				Five week users			
	Age 30-39	4.24	(1.57,11.42)		Less than half pack	1.72	(1.23,2.44)
	Age 40-49	5.07	(1.91,13.45)		Unemployed	1.79	(1.33,2.38)
	Age 50+	8.37	(3.32,21.09)		No PTSD[a]	1.16	(0.88,1.56)

[a]No PTSD refers to screening negative for Post-Traumatic Stress Disorder.
